# Supplementary material for: DNA/RNA heteroduplex oligonucleotide technology for regulating lymphocytes in vivo
Source: Nat Commun. 2021 Dec 22;12:7344. doi: 10.1038/s41467-021-26902-8 (PMC8695577; doi:10.1038/s41467-021-26902-8)
Supplement: Supplementary file 2 — Supplementary Information [file 41467_2021_26902_MOESM2_ESM.pdf]

DNA/RNA heteroduplex oligonucleotide technology for regulating lymphocytes in vivo

Authors: Masaki Ohyagi[1, 2], Tetsuya Nagata[1, 2]*, Kensuke Ihara[3], Kie Yoshida-Tanaka[1, 2], Rieko Nishi[1, 2], Haruka Miyata[1, 2], Aya Abe[1, 2], Yo Mabuchi[4], Chihiro Akazawa[4], Takanori Yokota[1, 2]*

[1] Department of Neurology and Neurological Science, Graduate School of Medical and Dental Sciences, Tokyo Medical and Dental University, Tokyo, Japan

[2] Center for Brain Integration Research, Tokyo Medical and Dental University, Tokyo, Japan

[3] Department of Bio-informational Pharmacology, Medical Research Institute, Tokyo Medical and Dental University, Tokyo, Japan

[4] Department of Biochemistry and Biophysics, Graduate School of Medical and Dental Sciences, Tokyo Medical and Dental University, Tokyo, Japan

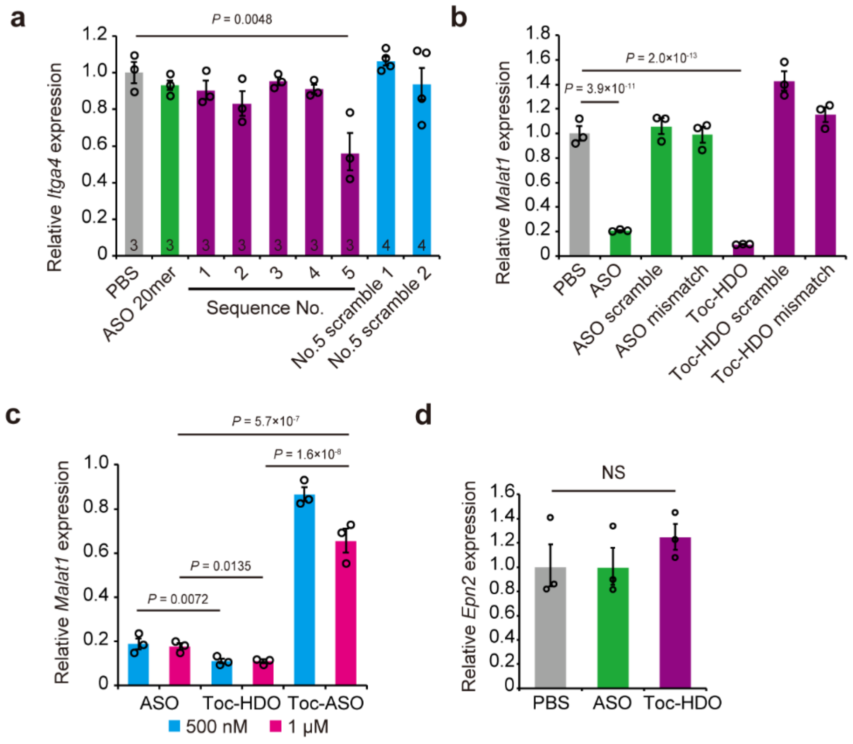

Supplementary Figure 1. Screening ASO sequences for *Itga4* mRNA and efficient inhibition of target gene expression by Toc-HDO treatment in mouse primary T cells in vitro. a Screening ASO sequences that efficiently reduce mouse *Itga4* mRNA expression in primary T cells measured by quantitative RT-PCR analyses 24 h after treatment with 500 nM ASO without any transfection reagents. Numbers at the bottom of each graph indicate samples per group. The ASO 20mer sequence was reported to knockdown *Itga4* mRNA expression in a mouse disease model. **b** Target *Malat1* RNA levels measured by RT-PCR in primary T cells 24 h after treatment with 1 µM of the indicated oligonucleotides targeted to *Malat1* without any transfection reagents ($n = 3$ for each group). **c** Quantitative RT-PCR analyses of *Malat1* RNA levels in primary T cells, which were incubated with *Malat1*-targeting ASO, Toc-HDO, or Toc-ASO at the indicated concentrations for 24 h without any transfection reagents ($n = 3$ for each group). **d** *Epn2* mRNA levels measured by quantitative RT-PCR in primary T cells 24 h after treatment with 1 µM *Itga4*-targeting Toc-HDO, ASO, and PBS alone ($n = 3$ for each group). NS, not significant. Data are normalized to *Gapdh* mRNA levels and are expressed as mean values ± s.e.m. Data represent at least two independent experiments. *P* values were calculated using one-way ANOVA with Holm's post-test. Source data are provided as a Source Data file.

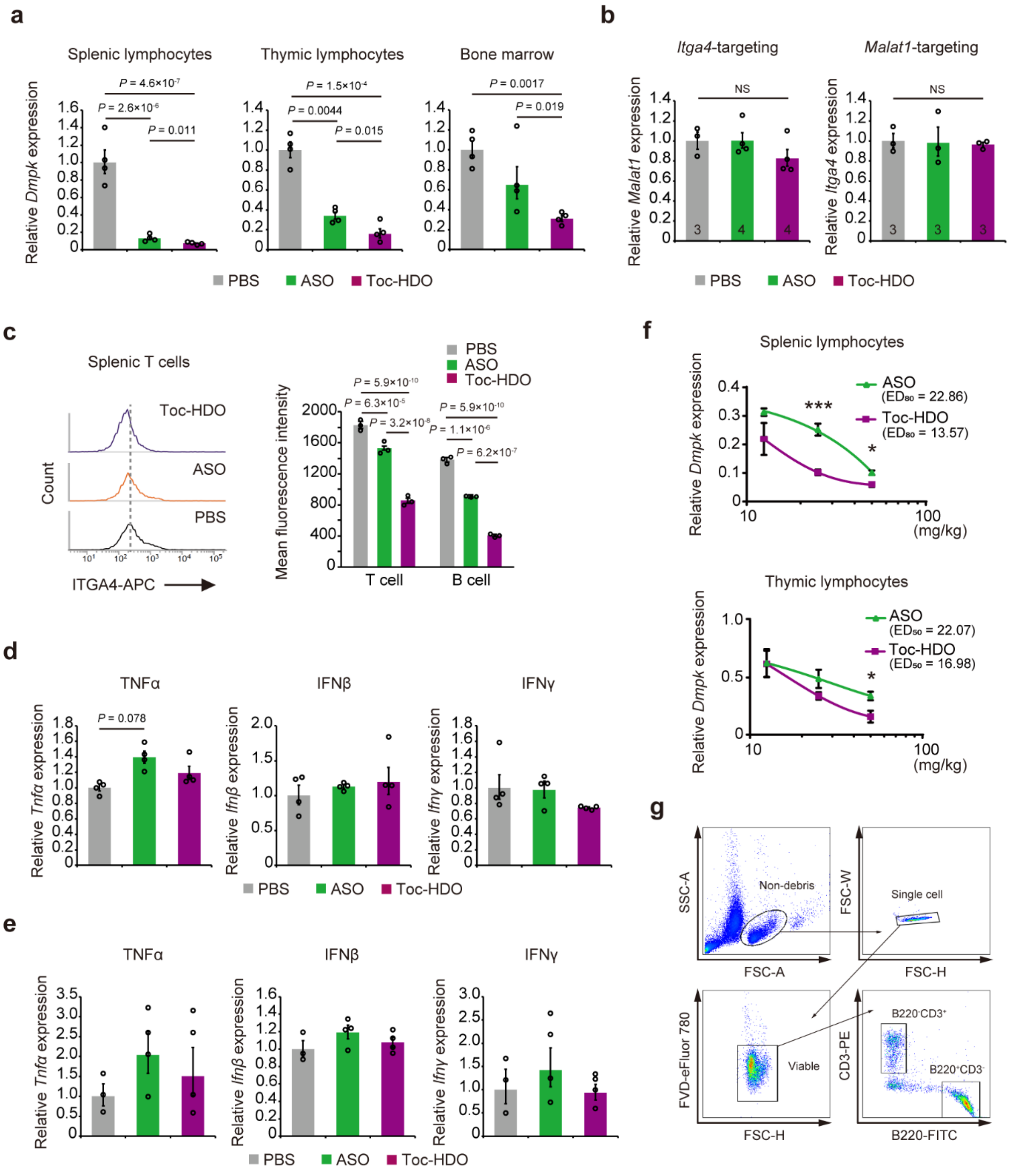

Supplementary Figure 2. Target gene and protein expression, and adverse immune stimulatory effects are inhibited by intravenous administration of Toc-HDO targeting endogenous genes in mouse lymphocytes in vivo. a Target *Dmpk* mRNA levels measured by quantitative RT-PCR in mouse lymphocytes in various tissues 72 h after intravenous injection of 50 mg/kg Toc-HDO, ASO, or PBS alone ($n = 4$ for each group). **b** Quantitative RT-PCR analyses of *Malat1* RNA levels in mouse lymph node lymphocytes 72 h after intravenous injection of 50 mg/kg *Itga4*-targeting Toc-HDO, ASO, or PBS alone (left), and *Itga4* mRNA levels in mouse lymph node lymphocytes 72 h after intravenous injection of 50 mg/kg *Malat1*-targeting Toc-

HDO, ASO, or PBS alone (right). Numbers at the bottom of each graph indicate mice per group. NS, not significant. **c** ITGA4 protein expression determined by flow cytometry 5 days after intravenous administration of 50 mg/kg Toc-HDO, ASO, or PBS alone (*n* = 3 for each group). **d**, **e** Adverse immune stimulatory effects of intravenously-administered 50 mg/kg Toc-HDO or ASO targeting *Dmpk* (**d**; *n* = 4 for each group) or *Itga4* (**e**; PBS, *n* = 3; ASO, *n* = 4; Toc-HDO, *n* = 4) in mouse lymphocytes. *Tnfα*, *tumor necrosis factor-α*; *Ifnβ*, *interferon-β*; *Ifnγ*, *interferon-γ*. **f** Dose dependence study of intravenously-administered Toc-HDO or ASO targeting *Dmpk* in mouse splenic and thymic lymphocytes 72 h after intravenous injection of Toc-HDO, ASO, or PBS alone (*n* = 4 for each group). **g** The sorting/gating strategy. Cells were identified first on FSC/SSC plots; dead cells were subsequently gated away using a Fixable Viability Dye eFluor 780 (eBioscience); live cells were plotted against CD3 and CD45R/B220 for the determination of each positive cells. Quantitative RT-PCR data are normalized to *Gapdh* mRNA levels and are expressed as mean values ± s.e.m. Data represent at least two independent experiments. *P* values were calculated using one-way ANOVA with Holm's post-test. Source data are provided as a Source Data file.

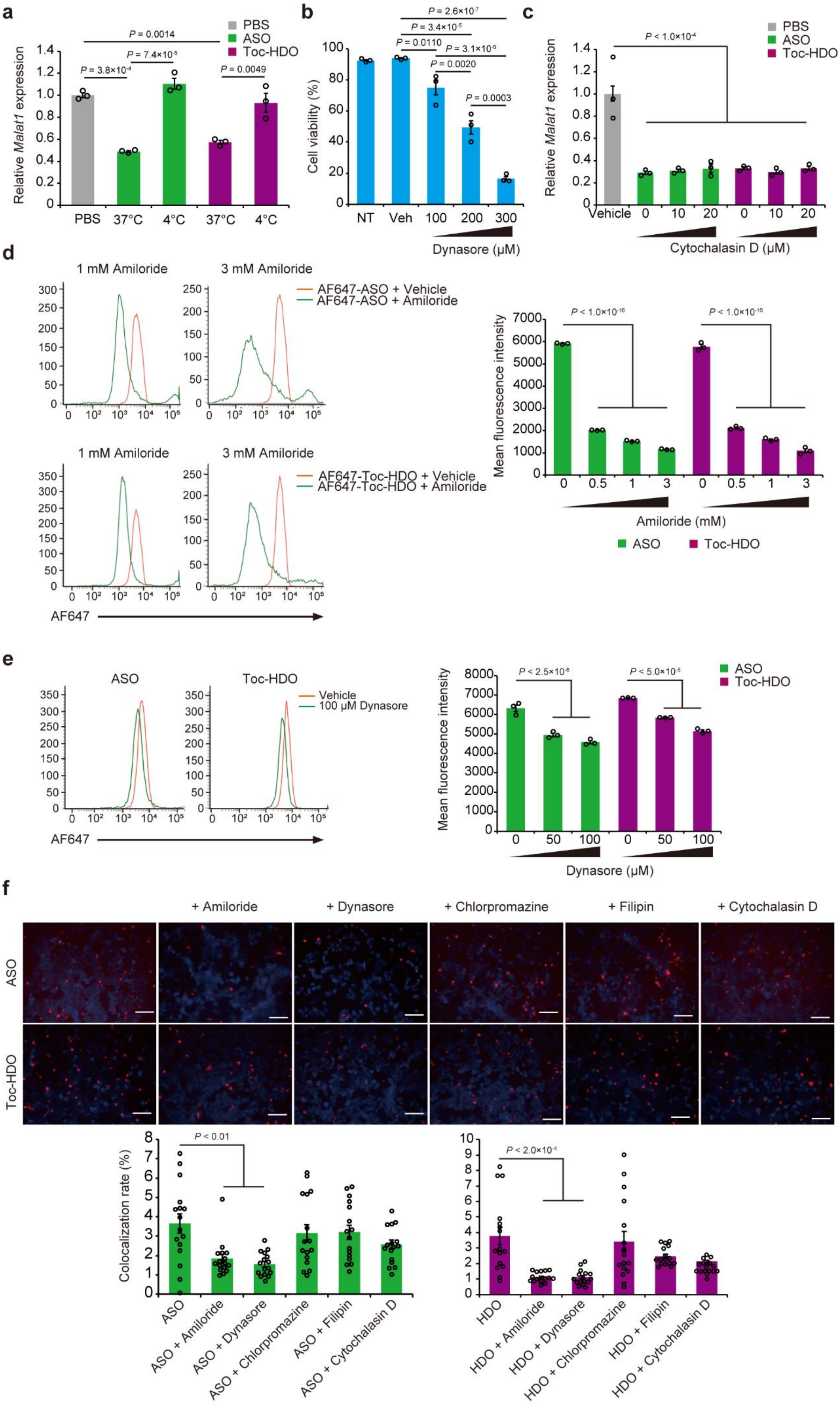

a
PBS
ASO
Toc-HDO
P = 0.0014
P = 7.4×10⁻⁵
P = 3.8×10⁻⁴
P = 0.0049
Relative Malat1 expression
PBS 37°C 4°C 37°C 4°C
b
P = 2.6×10⁻⁷
P = 3.4×10⁻⁵
P = 0.0110
P = 3.1×10⁻⁶
P = 0.0020
P = 0.0003
Cell viability (%)
NT Veh 100 200 300
Dynasore (μM)
c
PBS
ASO
Toc-HDO
P < 1.0×10⁻⁴
Relative Malat1 expression
Vehicle 0 10 20 0 10 20
Cytochalasin D (μM)
d
1 mM Amiloride
3 mM Amiloride
AF647-ASO + Vehicle
AF647-ASO + Amiloride
AF647-Toc-HDO + Vehicle
AF647-Toc-HDO + Amiloride
AF647
P < 1.0×10⁻¹⁶
Mean fluorescence intensity
0 0.5 1 3 0 0.5 1 3
Amiloride (mM)
ASO
Toc-HDO
e
ASO
Toc-HDO
Vehicle
100 μM Dynasore
AF647
P < 2.5×10⁻⁶
P < 5.0×10⁻⁵
Mean fluorescence intensity
0 50 100 0 50 100
Dynasore (μM)
ASO
Toc-HDO
f
+ Amiloride
+ Dynasore
+ Chlorpromazine
+ Filipin
+ Cytochalasin D
ASO
Toc-HDO
P < 0.01
Colocalization rate (%)
ASO
ASO + Amiloride
ASO + Dynasore
ASO + Chlorpromazine
ASO + Filipin
ASO + Cytochalasin D
P < 2.0×10⁻⁴
HDO
HDO + Amiloride
HDO + Dynasore
HDO + Chlorpromazine
HDO + Filipin
HDO + Cytochalasin D

Supplementary Figure 3. Endocytosis inhibitor reduce Toc-HDO cellular uptake in mouse T cells in vitro. a Effects of inhibition by cool stimulation on gene silencing of Toc-HDO and ASO measured by quantitative RT-PCR analyses of *Malat1* RNA levels in the EL4 cells incubated with 500 nM Toc-HDO or ASO at the indicated temperature for 4h, washed, and cultured for another 20 h (n = 3 for each group). **b** Percent of cell viability of EL4 cells measured by flow cytometry analysis using fixable viability dyes staining after 1 h treatment with indicated dose of dynasore (n = 3 for each group). **c** Effects of phagocytosis inhibitor on gene silencing of Toc-HDO and ASO. Target *Malat1* RNA levels in the EL4 cells treated with cytochalasin D, followed by treatment with Toc-HDO or ASO. EL4 cells were incubated with 10 or 20 µM cytochalasin D for 1 h and treated with 500 nM Toc-HDO or ASO, washed, and cultured for another 20 h before RNA isolation (n = 3 for each group). **d**, **e** Dose dependent inhibition of Alexa Fluor 647 (AF647)-labeled Toc-HDO or ASO cellular internalization in the EL4 cells treated with amiloride (**d**; n = 3 for each group) or dynasore (**e**; n = 3 for each group) analyzed by flow cytometry. **f** Representative images and quantitative data of AF647-labeled Toc-HDO or ASO internalized by EL4 cells treated with each inhibitor (n = 16 for each group). Sections were stained with DAPI. Red, AF647; blue, DAPI. Scale bars, 50 µm. Quantitative RT-PCR data shown are relative to *Gapdh* mRNA levels. Data are expressed as mean values ± s.e.m. and represent at least two independent experiments. P values were calculated using one-way ANOVA with Holm's post-test. Source data are provided as a Source Data file.

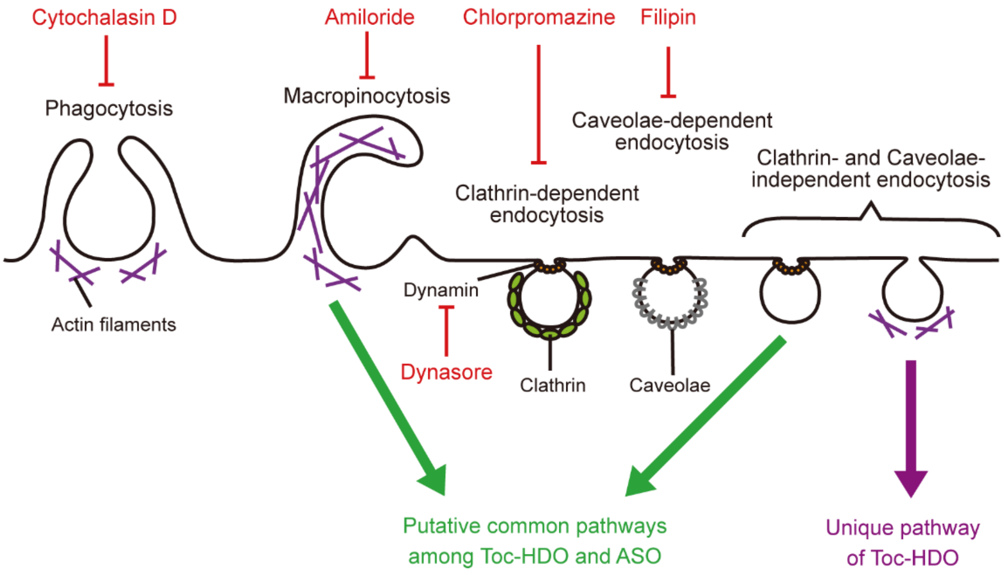

Supplementary Figure 4. Illustration of putative endocytosis pathways for Toc-HDO and ASO. A schematic representation of the proposed gymnotic delivery mechanisms for Toc-HDO in mouse lymphocytes in vitro. Multiple pathways regulate endocytosis into cells. The micropinocytosis and dynamin-dependent, clathrin- and caveolae-independent pathways are the possible common pathways between Toc-HDO and ASO, whereas the clathrin- and caveolae-independent pathway in which dynamin is not involved, may be unique to Toc-HDO. The compounds indicated in red are the endocytosis inhibitors used in this study. ASO, antisense oligonucleotide; Toc-HDO, α-tocopherol conjugated DNA/RNA heteroduplex oligonucleotide.

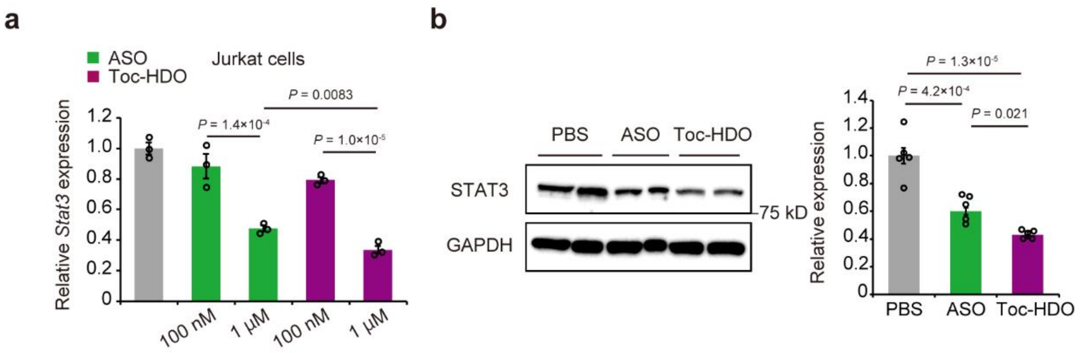

Supplementary Fig 5. Gene silencing and protein expression are inhibited by Toc-HDO targeting endogenous gene in human cultured T cells. a Target *Stat3* mRNA levels measured by quantitative RT-PCR analyses in Jurkat cells 24 h after treatment with 100 nM or 1 μM of *Stat3*-targeting Toc-HDO, ASO, or PBS alone without any transfection reagents (n = 3 for each group). **b** STAT3 protein expression determined by western blot in Jurkat cells after treatment with *Stat3*-targeting Toc-HDO or ASO for 24 h (n = 5 for each group). Quantitative RT-PCR data and band intensity shown are relative to *Gapdh* mRNA and GAPDH protein levels, respectively. Data are expressed as mean values ± s.e.m. and represent at least two independent experiments. P values were calculated using one-way ANOVA with Holm's post-test. Source data are provided as a Source Data file.

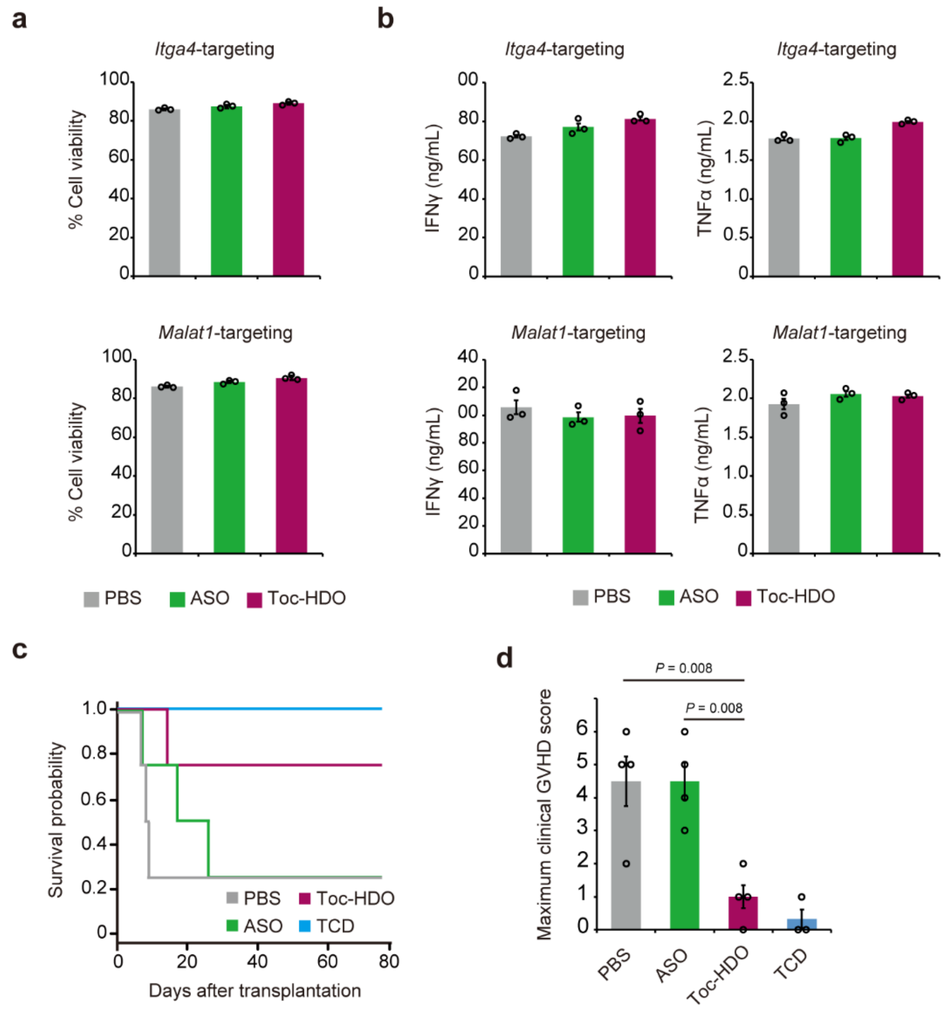

Supplementary Figure 6. Toc-HDO-mediated suppression of Itga4 prevents graft versus host disease (GVHD) with no effect on cell viability and dysfunctional cytokine production. **a** Percent of cell viability measured by flow cytometry analysis using fixable viability dyes staining after 24 h incubation with 1 μM of *Itga4*- or *Malat1*-targeting Toc-HDO, ASO, or PBS alone (*n* = 3 for each group). **b** ELISA analysis of IFNγ (left) and TNFα (right) production by primary T cells treated with 1 μM of *Itga4*- (upper) or *Malat1*-targeting (lower) Toc-HDO or ASO for 24 h, followed by 4 h incubation with PMA and ionomycin (*n* = 3 for each group). **c**, **d** Survival curve (**c**) and maximum clinical GVHD score (**d**) of BALB/c recipients after transplant with T cell-depleted C57BL/6J bone marrow (TCD) and splenic T cells treated with *Itga4*-targeting Toc-HDO, ASO, or PBS (PBS, *n* = 4; ASO, *n* = 4; Toc-HDO, *n* = 4; TCD, *n* = 3). Data are expressed as mean values ± s.e.m. and represent at least two independent experiments. *P* values were calculated using one-way ANOVA with Holm's post-test (**a**, **b**, and **d**) or log-rank test for survival curves (**d**). Source data are provided as a Source Data file.

Supplementary Table 1. ASO sequences targeting mouse *Itga4* mRNA for in vitro experiments.

ASO 20mer	5' **C**^**C**^**G**^**C**^**A**^g^c^c^a^t^g^c^g^c^t^**C**^**T**^**T**^**G**^**G** 3'
No. 1	5' **C**^**C**^**G**^c^a^g^c^c^a^t^g^c^g^**C**^**T**^**C** 3'
No. 2	5' **C**^**G**^**C**^a^g^c^c^a^t^g^c^g^c^**T**^**C**^**T** 3'
No. 3	5' **G**^**C**^**A**^g^c^c^a^t^g^c^g^c^t^**C**^**T**^**T** 3'
No. 4	5' **C**^**A**^**G**^c^c^a^t^g^c^g^c^t^c^**T**^**T**^**G** 3'
No. 5	5' **A**^**G**^**C**^c^a^t^g^c^g^c^t^c^t^**T**^**G**^**G** 3'
No. 5 scramble 1	5' **G**^**C**^**G**^a^c^g^c^g^t^t^c^a^c^**T**^**C**^**T** 3'
No. 5 scramble 2	5' **G**^**C**^**G**^g^c^g^a^t^c^t^a^t^g^**C**^**T**^**C** 3'

Lowercase letters present DNA, bold uppercase letters represent locked nucleic acid (LNA), capital C denotes LNA 5-methylcytosine, and caret represents phosphorothioate linkages.

Supplementary Table 2. Antibodies used in this study.

Antibodies	Company	Catalog#	Application	Dilution
Hamster anti-Mouse CD3	BD Pharmingen	553058	culture	1 µg/ml
Hamster anti-Mouse CD28	BD Pharmingen	553295	culture	2 µg/ml
PE Rat anti-Mouse CD3	BioLegend	100205	Flow	1:200
PE-Cy7 Rat anti-Mouse CD45R/B220	BioLegend	103221	Flow	1:200
FITC Rat anti-Mouse CD45R/B220	BioLegend	103205	Flow	1:200
APC Rat anti-Mouse CD49d	BioLegend	103621	Flow	1:200
Integrin α4 Rabbit IgG	Cell Signaling	8440	WB	1:1,000
STAT3 Mouse IgG	Cell Signaling	9139	WB	1:1,000
GAPDH monoclonal antibody, Peroxidase Conjugated	Wako	015-25473	WB	1:10,000
Rabbit anti-Mouse CD4	NOVUS Biologicals	NBP1-19371	IHC	1:200
Rat anti-Mouse myelin basic protein	Abcam	Ab7349	IF	1:800
Rabbit anti-Mouse Iba1	Wako	019-19741	IF	1:400
Goat anti-Rat IgG-Alexa Fluor 488	Invitrogen	A11006	IF	1:200
Goat anti-Rabbit IgG-Alexa Fluor 647	Invitrogen	A21244	IF	1:200

Abbreviation: Flow, Flow cytometry; IF, immunofluorescence; IHC, immunohistochemistry; WB, western blot

Supplementary Table 3. Concentrations of endocytosis pathway inhibitors used in this study.

Inhibitors	Concentrations used	50% inhibitory concentration (IC50)
Amiloride	1, 3 mM	200–500 μM [25,26]
Chlorpromazine	5, 10, 30 μM	17.4 μM [30]
Cytochalasin D	10 μM	1.4 μM [34]
Dynasore	10, 50, 100 μM	15 μM [31]
Filipin	1, 2, 3 μM	0.4–2 μM [32,33]
