# Supplementary material for: DNA/RNA heteroduplex oligonucleotide technology for regulating lymphocytes in vivo
Source: Nat Commun. 2021 Dec 22;12:7344. doi: 10.1038/s41467-021-26902-8 (PMC8695577; doi:10.1038/s41467-021-26902-8)
Supplement: Supplementary file 3 — Reporting Summary [file 41467_2021_26902_MOESM3_ESM.pdf]

nature portfolio

Corresponding author(s): Yokota T and Nagata T
Last updated by author(s): Sep 30, 2021

Reporting Summary

Nature Portfolio wishes to improve the reproducibility of the work that we publish. This form provides structure for consistency and transparency in reporting. For further information on Nature Portfolio policies, see our Editorial Policies and the Editorial Policy Checklist.

Statistics

For all statistical analyses, confirm that the following items are present in the figure legend, table legend, main text, or Methods section.

n/a	Confirmed	
☐	☒	The exact sample size (n) for each experimental group/condition, given as a discrete number and unit of measurement
☐	☒	A statement on whether measurements were taken from distinct samples or whether the same sample was measured repeatedly
☐	☒	The statistical test(s) used AND whether they are one- or two-sided<br>*Only common tests should be described solely by name; describe more complex techniques in the Methods section.*
☒	☐	A description of all covariates tested
☐	☒	A description of any assumptions or corrections, such as tests of normality and adjustment for multiple comparisons
☐	☒	A full description of the statistical parameters including central tendency (e.g. means) or other basic estimates (e.g. regression coefficient) AND variation (e.g. standard deviation) or associated estimates of uncertainty (e.g. confidence intervals)
☐	☒	For null hypothesis testing, the test statistic (e.g. F, t, r) with confidence intervals, effect sizes, degrees of freedom and P value noted<br>*Give P values as exact values whenever suitable.*
☒	☐	For Bayesian analysis, information on the choice of priors and Markov chain Monte Carlo settings
☒	☐	For hierarchical and complex designs, identification of the appropriate level for tests and full reporting of outcomes
☒	☐	Estimates of effect sizes (e.g. Cohen's d, Pearson's r), indicating how they were calculated

Our web collection on statistics for biologists contains articles on many of the points above.

Software and code

Policy information about availability of computer code

Data collection	Light cycler 480 software release 1.5.1.62 SP3<br>BD FACSuite ver1.2<br>BD FACSDiva ver8.0<br>FlowJo ver10.6
Data analysis	FIJI image processing software (NIH) – v2.0.0-rc-59/1.51n<br>Microsoft Excel 2016 MSO (16.0.4266.1001)<br>Graphpad prism 8.3.0

For manuscripts utilizing custom algorithms or software that are central to the research but not yet described in published literature, software must be made available to editors and reviewers. We strongly encourage code deposition in a community repository (e.g. GitHub). See the Nature Portfolio guidelines for submitting code & software for further information.

Data

Policy information about availability of data

All manuscripts must include a data availability statement. This statement should provide the following information, where applicable:

- Accession codes, unique identifiers, or web links for publicly available datasets
- A description of any restrictions on data availability
- For clinical datasets or third party data, please ensure that the statement adheres to our policy

The data are available from the corresponding authors upon reasonable request. Source data are provided as a Source Data file.

Field-specific reporting

Please select the one below that is the best fit for your research. If you are not sure, read the appropriate sections before making your selection.

[x] Life sciences [ ] Behavioural & social sciences [ ] Ecological, evolutionary & environmental sciences

For a reference copy of the document with all sections, see nature.com/documents/nr-reporting-summary-flat.pdf

Life sciences study design

All studies must disclose on these points even when the disclosure is negative.

Sample size	No sample size calculations were performed beforehand, but all experiments were repeated at least two times. Sample size was determined to be adequate based on previously published researches.
Data exclusions	No samples were excluded from the analysis.
Replication	Experiments were repeated with similar results at least two times with independent biological replicates. Number of reliable reproductions of each experimental finding is indicated in each figure legend.
Randomization	Allocation of animals to the different groups was random by trained researchers performing each experiment.
Blinding	No experiment, except for experimental autoimmune encephalomyelitis, has been blinded. Clinical score of experimental autoimmune encephalomyelitis was assessed by a blinded researcher.

Reporting for specific materials, systems and methods

We require information from authors about some types of materials, experimental systems and methods used in many studies. Here, indicate whether each material, system or method listed is relevant to your study. If you are not sure if a list item applies to your research, read the appropriate section before selecting a response.

Materials & experimental systems

n/a	Involved in the study	
[ ]	[x]	Antibodies
[ ]	[x]	Eukaryotic cell lines
[x]	[ ]	Palaeontology and archaeology
[ ]	[x]	Animals and other organisms
[x]	[ ]	Human research participants
[x]	[ ]	Clinical data
[x]	[ ]	Dual use research of concern

Methods

n/a	Involved in the study	
[x]	[ ]	ChIP-seq
[ ]	[x]	Flow cytometry
[x]	[ ]	MRI-based neuroimaging

Antibodies

Antibodies used

FACS: Antibodies for flow cytometry were purchased from BioLegend and used at a concentration of 1:200.
Anti-CD3e-PE (BioLegend, clone 17A2, 100205)
Anti-CD45R/B220-PECy7 (BioLegend, clone RA3-6B2, #103221)
Anti-CD45R/B220-FITC (BioLegend, clone RA3-6B2, #103205)
Anti-CD49d-APC (BioLegend, clone R1-2, #103621)
Other:
Anti-CD4 (NOVUS Biologicals, #NBP1-19371)
Anti-myelin basic protein (Abcam, # ab40390)
Anti-Iba1 (Wako, #019-19741)
Anti-CD3 antibody (BD Pharmingen, clone 145-2C11, #553058)
Anti-CD28 antibody (BD Pharmingen, clone 37.51, #557393)
Integrin α4 Rabbit IgG (Cell Signaling, clone D2E1, #8440)
STAT3 Mouse IgG (Cell Signaling, clone 124H6, #9139)
Anti-GAPDH monoclonal antibody, Peroxidase Conjugated (Wako, clone 5A12, #015-25473)
Goat anti-Rat IgG-Alexa Fluor 488 (Invitrogen, #A11006)
Goat anti-Rabbit IgG-Alexa Fluor 647 (Invitrogen, #A21244)

Validation

All antibodies were validated by the manufacturer; detailed validation analysis and relevant literatures are provided on the company website for the products used in this study.

Anti-CD3e-PE (https://www.biolegend.com/ja-jp/products/pe-anti-mouse-cd3-antibody-47?GroupID=BLG242)
Anti-CD45R/B220-PECy7 (https://www.biolegend.com/ja-jp/search-results/pe-cyanine7-anti-mouse-human-cd45r-b220-antibody-1930)
Anti-CD45R/B220-FITC (https://www.biolegend.com/ja-jp/products/fitc-anti-mouse-human-cd45r-b220-antibody-445?GroupID=GROUP658)
Anti-CD49d-APC (https://www.biolegend.com/ja-jp/products/apc-anti-mouse-cd49d-antibody-11893?GroupID=BLG10488)
Anti-CD4 (https://www.novusbio.com/products/cd4-antibody_nbp1-19371)
Anti-myelin basic protein (https://www.abcam.co.jp/myelin-basic-protein-antibody-ab40390.html)
Anti-Iba1 (https://labchem-wako.fujifilm.com/us/product/detail/W01W0101-1974.html)
Anti-CD3 antibody (https://www.bdbiosciences.com/en-us/products/reagents/flow-cytometry-reagents/research-reagents/single-color-antibodies-ruo/purified-hamster-anti-mouse-cd3e.553058)
Anti-CD28 antibody (https://www.bdbiosciences.com/ja-jp/products/reagents/flow-cytometry-reagents/research-reagents/single-color-antibodies-ruo/purified-hamster-anti-mouse-cd28.557393)
Integrin α4 Rabbit IgG (https://www.cellsignal.jp/products/primary-antibodies/integrin-a4-d2e1-xp-rabbit-mab/8440?Ns=product.currentLot.numberOfApplications%7C1&N=4294967206&Nrpp=200&fromPage=plp)
STAT3 Mouse IgG (https://www.cellsignal.jp/products/primary-antibodies/stat3-124h6-mouse-mab/9139)
Anti-GAPDH monoclonal antibody, Peroxidase Conjugated (https://labchem-wako.fujifilm.com/asia/product/detail/W01W0101-2547.html)
Goat anti-Rat IgG-Alexa Fluor 488 (https://www.thermofisher.com/antibody/product/Goat-anti-Rat-IgG-H-L-Cross-Adsorbed-Secondary-Antibody-Polyclonal/A-11006)
Goat anti-Rabbit IgG-Alexa Fluor 647 (https://www.thermofisher.com/antibody/product/Goat-anti-Rabbit-IgG-H-L-Cross-Adsorbed-Secondary-Antibody-Polyclonal/A-21244)

Eukaryotic cell lines

Policy information about cell lines

Cell line source(s)	EL4 (ATCC TIB-39) and Jurkat (ATCC E6-1) from ATCC.
Authentication	No authentication was performed for EL4 and Jurkat.
Mycoplasma contamination	No test for mycoplasma contamination was performed.
Commonly misidentified lines (See ICLAC register)	No commonly misidentified cell lines were used.

Animals and other organisms

Policy information about studies involving animals; ARRIVE guidelines recommended for reporting animal research

Laboratory animals	Male and female C57BL/6J mice (6–10 weeks old) and male BALB/c (10 weeks old) were purchased from the Charles River Laboratories. Animals were housed under specific pathogen-free conditions (temperature: 18–24°C; humidity: 40–70%) in a day-night controlled light cycle, provided with food and water ad libitum.
Wild animals	Study did not involve wild animals.
Field-collected samples	Study did not involve samples collected from the field.
Ethics oversight	The experimental procedures used in this study were approved by the Institutional Animal Care and Use Committee of Tokyo Medical and Dental University (No. 0170179A).

Note that full information on the approval of the study protocol must also be provided in the manuscript.

Flow Cytometry

Plots

Confirm that:

- [x] The axis labels state the marker and fluorochrome used (e.g. CD4-FITC).
- [x] The axis scales are clearly visible. Include numbers along axes only for bottom left plot of group (a 'group' is an analysis of identical markers).
- [x] All plots are contour plots with outliers or pseudocolor plots.
- [x] A numerical value for number of cells or percentage (with statistics) is provided.

Methodology

Sample preparation	Samples were prepared as described in Methods section.<br>Single cell suspensions were prepared from spleen and thymus tissues by mechanical dissociation using 70 µm nylon mesh, and diluted with an equal volume of sterile PBS. Peripheral blood and the diluted cell suspensions were carefully overlaid on three volumes of Lymphocyte Separation Medium 1077 in 15 ml tubes and centrifuged at 400 × g for 40 min without brakes.

	Mouse lymphocytes were removed from the liquid/medium interface and washed three times with 0.1% BSA in PBS. The cells were then washed and suspended in PBS with 2% bovine serum albumin and 0.05% sodium azide with antibodies to cell surface markers.
Instrument	BD FACSVerse, BD FACS Aria II.
Software	BD FACSuite ver1.2, FACSDiva ver 8.0, and FlowJo v10.6 software were used for acquisition and analysis of flow cytometry data.
Cell population abundance	At least 50,000 cells per sample were recorded using appropriate negative control during FACS analyses. Purity was determined by post-sort check.
Gating strategy	The sorting/gating strategy is presented in Supplementary Fig. 2g. Cells were identified first on FSC/SSC plots; dead cells were subsequently gated away using a Fixable Viability Dye eFluor 780 (eBioscience); live cells were plotted against CD3 and CD45R/B220 for the determination of each positive cells.

☒ Tick this box to confirm that a figure exemplifying the gating strategy is provided in the Supplementary Information.
